# Double V-Y Island Pedicle Flap for Dorsal Hand Reconstruction Following Mohs Micrographic Surgery

**DOI:** 10.7759/cureus.45314

**Published:** 2023-09-15

**Authors:** Neelesh P Jain, Brett C Neill, Christopher Garvey, Justin J Leitenberger

**Affiliations:** 1 Dermatology, University of Connecticut, Farmington, USA; 2 Dermatology, Oregon Health & Science University, Portland, USA

**Keywords:** melanoma in situ, dermatology, skin cancer, hand reconstructive surgery, reconstructive surgery, dermatologic surgery, mohs micrographic surgery

## Abstract

Medium to large defects on the dorsal hand pose a reconstructive challenge following dermatologic surgery. Repairs in this location can be complicated by a paucity of adjacent tissue reservoirs, competing tension vectors, thin cutaneous tissue, and superficial tendons and vasculature. In such cases, a double V-Y island pedicle flap is an effective reconstructive solution. It preserves hand function, harnesses local tissue with a robust blood supply, facilitates complete closure, and provides skin that closely matches the original’s color and texture. Here, we present the repair of a medium to large dorsal hand defect after Mohs micrographic surgery for melanoma in situ, using a double V-Y island pedicle flap.

## Introduction

Dermatologic surgery on the dorsal hand may lead to defects with minimal surrounding skin laxity or adjacent tissue reservoirs to use in a repair. Melanoma in situ in sun-damaged skin is particularly challenging as it often requires larger margins with subclinical extension peripherally [1]. Anatomically, the dorsal hand has a thin dermis and minimal subcutaneous fat, and defects may easily expose extensor tendons and superficial neurovascular bundles. In wounds healed by secondary intent, desiccation of exposed tendons can lead to contraction and functional disability, while exposed superficial vessels are prone to injury and bleeding [2]. Linear closure of medium to large dorsal hand defects is impeded due to a lack of surrounding tissue laxity. Skin grafting is often employed for large wounds on the dorsal hand. However, it may necessitate extended immobilization or bandaging of the hand and carries risks of graft failure, contraction, and skin complexion mismatch [3,4]. Rhombic, bilobed, and rotation flaps require local tissue reservoirs and have limited mobility due to their cutaneous connections, making them suboptimal for closing large defects [5]. A fasciocutaneous sliding flap can achieve great results for dorsal hand defects but is generally more suitable for medium-sized defects [6]. The reverse radial forearm flap is another single-stage repair option. However, it requires extensive surgery to transfer distant tissue, may compromise blood flow due to ligation of the proximal radial artery, necessitates a skin graft to close the secondary defect, and is at risk of causing long-term joint stiffness [7,8]. Herein, we describe the challenge of reconstructing a medium to large defect on the dorsal hand, resulting from Mohs surgery for melanoma in situ, utilizing a double V-Y island pedicle flap.

## Case presentation

**Figure 1:**
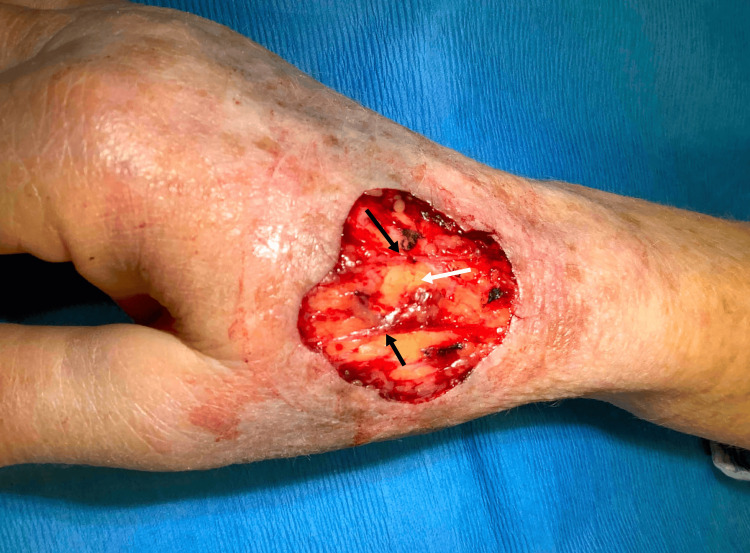
Postoperative Mohs defect, measuring 4.0 x 3.0 cm, on the dorsal right hand. The wound extends down to the level of the subcutaneous fat in the dorsal intermediate lamina (white arrow), with exposure of intact superficial vasculature (black arrows).

A 68-year-old female was referred for Mohs micrographic surgery of a biopsy-proven melanoma in situ on the dorsal right hand. The tumor appeared as a brown patch measuring 2.6 x 1.5 cm. Peripheral and deep margins were excised and examined using en-face frozen sections stained with MART-1, achieving tumor clearance after four stages. The resulting 4.0 x 3.0 cm defect extended down to the subcutaneous fat in the dorsal intermediate lamina, just superior to the plane in which the extensor tendons reside, with exposure of superficial vasculature (Figure 1).

**Figure 2:**
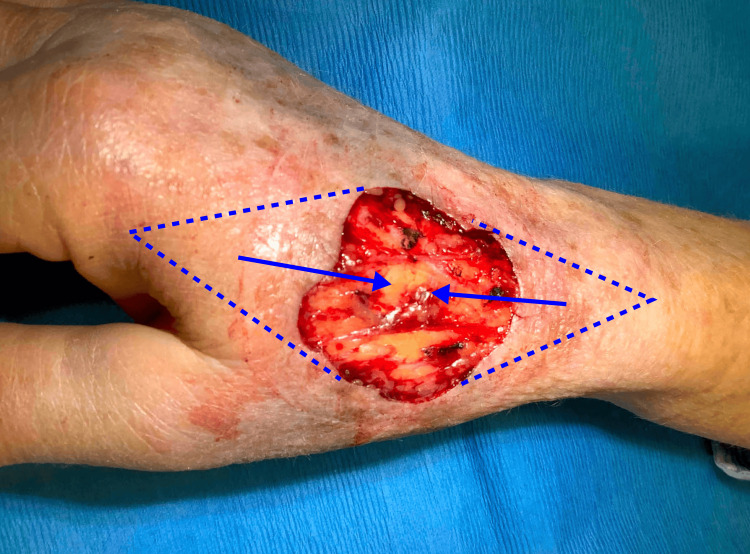
Design of a double V-Y island pedicle flap (dashed blue lines). Solid blue arrows depict the direction of flap movement.

The authors opted to repair the defect with two V-Y island pedicle flaps recruited from tissue proximal and distal to the primary defect. Triangles were drawn with their bases extending from the proximal and distal edges of the wound (Figure 2).

**Figure 3:**
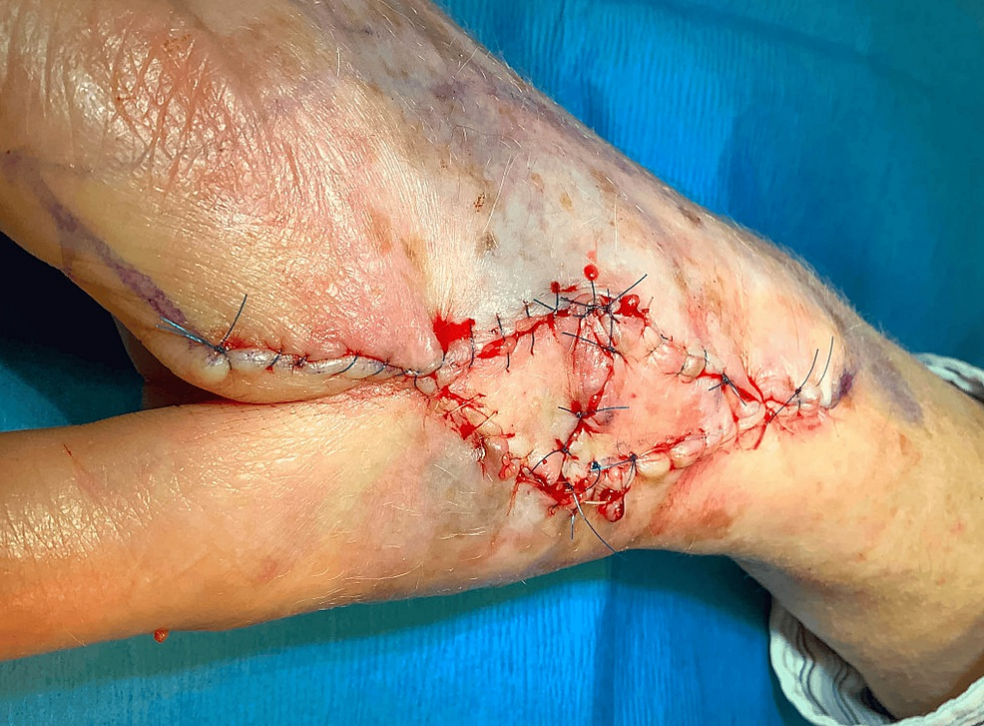
Final sutured flap.

Incisions were carried out down to the subcutaneous fat, allowing for elevation of each triangular flap and further dissection of the underlying pedicles to improve mobility, ultimately leaving approximately 60% of the original pedicle intact. Peripheral edges were then widely undermined at the level of the subcutaneous fat and the two triangular flaps were advanced toward the middle of the primary defect. Key stitches were placed, the flaps were inset, and secondary defects were closed with 4-0 glycolide/lactide copolymer buried vertical mattress sutures. The epidermal portion was approximated using 5-0 polypropylene and 5-0 fast-absorbing gut in a simple running and interrupted fashion. The final repair dimensions were 7.2 x 2.8 cm (Figure 3).

## Discussion

Small to medium-sized wounds on the dorsal hand can be left to heal via secondary intent, closed primarily in a linear fashion, or repaired via adjacent tissue transfer, all with excellent functional and cosmetic results [5]. In contrast, larger defects pose unique reconstructive challenges because of the risk of hand dysfunction due to scarring and contraction, finite skin available for primary closures, and high tension in multiple vectors [2,3]. Reconstructive options that require extended immobilization of the hand can delay patients in returning to their daily activities. For deeper defects, the risks of tendon desiccation and neurovascular compromise must also be considered [2]. Broadly speaking, reconstructive options for medium to large dorsal hand defects include healing by secondary intention, purse-string closure, linear repair, full or split-thickness grafts, advancement flaps, rotation flaps, and transposition flaps [5]. Healing by secondary intent is typically not an ideal option for larger defects on the dorsal hand due to long healing times, scar contraction, and poor cosmetic results [9]. Purse-string closures help reduce the size of a large wound but can result in excessively high tension on fragile thin skin, and circumferential pleating [9,10]. Linear repairs are also difficult due to high tension and potentially compromised articulation of the digits and wrist in the short postoperative period. Both full-thickness and split-thickness grafts can be good options to repair medium to large dorsal hand defects but require additional surgery to harvest from a donor site, longer immobilization, and remain at risk of graft failure [3-5]. Rotation flaps, bilobed flaps, and rhombic flaps suffer from a lack of adjacent tissue reservoirs and often require long incisions, sometimes with a back-cut that can reduce the flap’s blood supply [5]. Other options, including the fasciocutaneous sliding flap, may not allow for enough mobility to close larger defects [6]. The reverse radial forearm flap involves extensive additional surgery to recruit proximal tissue, ligate the radial artery, and fill the secondary defect with a skin graft [7,8].

**Figure 4:**
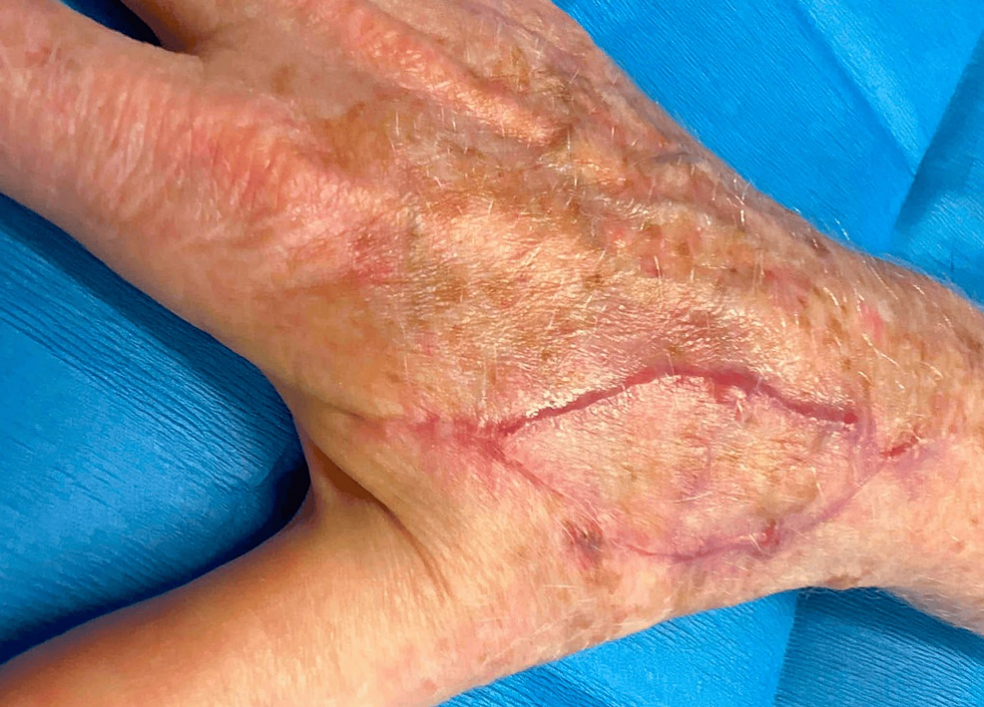
Patient’s dorsal right hand four months after repair.

In the case described, the authors opted for reconstruction with a double V-Y island pedicle flap. This is an excellent choice for medium to large dorsal hand defects as it allows maximal tissue preservation, redirection of primary defect tension vectors, transfers tissue with a robust vascular supply, avoids rotational forces that may reduce blood flow, preserves function, and optimizes cosmesis. While V-Y island pedicle flaps have typically been used for smaller defects, the use of a double V-Y island pedicle flap in this case allowed for coverage of a larger defect, and because the flap lacks a cutaneous connection, it benefitted from increased mobility compared to other options. This choice was also concordant with the patient’s preferences for a same-day repair without an extended period of immobilization. At a follow-up four months later, she had fully healed and was happy with both her functional and cosmetic results (Figure 4).

## Conclusions

Medium to large dorsal hand defects present a distinctive reconstructive challenge. Repair of the primary defect with a double V-Y island pedicle flap is a reliable and reproducible same-day repair with satisfactory functional and cosmetic outcomes. The presented case shows how this unique flap can be used to effectively reconstruct medium to large dorsal hand defects to achieve an excellent overall result.
